# Nicotinamide N-methyltransferase enhances paclitaxel resistance in ovarian clear cell carcinoma

**DOI:** 10.1007/s13577-025-01282-z

**Published:** 2025-08-25

**Authors:** Ryoko Kikuchi-Koike, Masaru Sakamoto, Yuko Sasajima, Yuko Miyagawa, Hiroshi Uozaki, Kenji Umayahara, Kei Hashimoto, Yuko Takahashi, Kazuki Takasaki, Chikara Kihira, Haruka Nishida, Takayuki Ichinose, Mana Hirano, Haruko Hiraike, Kazunori Nagasaka

**Affiliations:** 1 Department of Obstetrics and Gynecology, Teikyo University School of Medicine, 11-1 Kaga, Itabashi-Ku, Tokyo, Japan; 2 Department of Gynecology, Sasaki Foundation Kyoundo Hospital, Tokyo, Japan; 3 Department of Obstetrics and Gynecology, Jikei University School of Medicine, Tokyo, Japan; 4 Department of Pathology, Teikyo University School of Medicine, Tokyo, Japan

**Keywords:** Ovarian clear cell carcinoma, Chemoresistance, Paclitaxel, Nicotinamide N-methyltransferase, 2D-DIGE

## Abstract

Nicotinamide N-methyltransferase (NNMT) is an S-adenosyl-l-methionine (SAM)-dependent cytosolic enzyme, and a growing body of evidence suggest that it plays an essential role in cancer progression. Recently, NNMT has a role in methylation metabolism and tumorigenesis and was associated with a poor prognosis against numerous cancers. In addition, it has been reported that NNMT has been overexpressed in the stroma of advanced high-grade serous carcinoma and may contribute to decreased survival. This study aimed to identify novel biomarkers to predict resistance and investigate their clinicopathologic significance in paclitaxel-resistant advanced or recurrent ovarian clear cell carcinoma (OCCC). Fluorescence-labeled two-dimensional differential gel electrophoresis (2D-DIGE), immunohistochemical, and MASCOT analyses allowed us to identify the cytoplasmic metabolic enzyme NNMT. In cultured cell studies, NNMT protein expression was higher in paclitaxel-resistant OVMANA and OVTOKO cells than in paclitaxel-sensitive KK and ES-2 cells. Furthermore, although analysis of clinical tissue samples showed no association with poor prognosis in 7 individuals with low NNMT expression in the cytoplasm of OCCC cells, high expression of NNMT in the cytoplasm of OCCC cells may be associated with low sensitivity to paclitaxel in OCCC and may have prognostic implications. Therefore, targeting therapy to reduce cytoplasmic NNMT expression levels may increase the sensitivity of OCCC to paclitaxel.

## Introduction

Ovarian cancer is the fifth most common cause of cancer-related deaths in women after lung, breast, colorectal, and pancreatic cancers [1]. Ovarian clear cell carcinoma (OCCC) is a common histological type of epithelial ovarian cancer (EOC) in Asia. Women diagnosed with OCCC are usually younger and at an earlier stage of cancer than those with the common high-grade serous adenocarcinoma histology [2]. The overall prognosis of OCCC is good because most cases are identified during stage I of cancer [3]. However, advanced and recurrent disease is associated with a poor prognosis and resistance to standard treatment [4]. While serous adenocarcinoma, the most common type of ovarian cancer, responds well to anticancer drug therapy, ovarian clear cell carcinoma is known to have an abysmal prognosis due to the low efficacy of anticancer drug therapy. Although there are many patients with this type of ovarian clear cell carcinoma in Asia, the number of patients with this type of cancer is relatively small in Europe and the United States, and no progress has been made in developing therapeutic agents. Patients with OCCC show a lower response rate to paclitaxel and carboplatin treatment regimens than the non-OCCC ovarian cancer group [5]. Improving the response rate of patients with OCCC to paclitaxel and carboplatin regimens may improve the prognosis of patients with OCCC. Aoki et al. reported that taxane-based chemotherapy was effective in patients with OCCC that were positive for beta-tubulin III [6]. Ho et al. reported that restoration of HIN-1 expression reversed paclitaxel resistance in OCCC [7]. Yet, the molecules involved in the efficacy of taxane-based chemotherapy for OCCC have not been identified. A retrospective analysis of a multicenter, Phase 3, randomized, controlled trial (JGOG3017/GCIG) comparing TC (carboplatin and paclitaxel) and CPT-P (irinotecan and cisplatin) therapy in patients with recurrent or persistent OCCC found that patients with platinum-resistant relapse had a significantly shorter median post-progression survival (PPS) compared to those with platinum-sensitive relapse [8]. Combination therapy with gemcitabine, cisplatin, and bevacizumab (GP + Bev) has also been reported to be promising for advanced OCCC [9]. The identification of molecules involved in paclitaxel resistance in OCCC may provide an opportunity to improve the efficacy of paclitaxel and carboplatin regimens in OCCC. Therefore, we aimed to analyze the effect of paclitaxel treatment on the OCCC cell lines ES-2, KK, OVMANA, and OVTOKO, and the associated molecular changes using fluorescence-labeled two-dimensional differential gel electrophoresis (2D-DIGE).

## Materials and methods

### Cell lines and primary tumor samples

OVTOKO [10] and OVMANA [11] cell lines were obtained from the Japanese Collection of Research Bioresources (Osaka, Japan), ES-2 from the American Type Culture Collection (ATCC), KK from the National Defense Medical College [12], RMG1 from the Keio University [13]. ES-2 is a cell line exhibiting fibroblast-like morphology that was isolated from the ovary of a Black, 47-year-old, female human with clear cell carcinoma. This cell line was deposited to ATCC by BI Sikic and used in cancer research. Primary tumor samples were obtained during surgery from 39 patients treated at Teikyo University Hospital in Tokyo, with written consent obtained from each patient after approval was approved by the Clinical Ethics Committee of the Medical Faculty at Teikyo University (approval number: 13–003-4 on 22 October 2020). The study adheres to the provisions specified in the Declaration of Helsinki.

### Proliferation assay

The effects of paclitaxel on cell proliferation were evaluated using a WST-8 assay (Dojindo, Kumamoto, Japan). The assay can be used to evaluate cell viability in cell proliferation assays. WST-8 is reduced by dehydrogenase activity in cells to produce a water-soluble yellow-colored formazan dye formed by NADH produced in the mitochondria. The amount of formazan dye produced is directly proportional to the number of living cells. The protocol is described briefly as follows. Cells were seeded in 24-well plates and pre-incubated at 37 °C for 24 h to enable cell attachment. At 72 h after drug treatment, 50 μL of WST-8 solution was added to each well and incubated at 37 °C for 0.5–4 h (the reaction time was adjusted according to the cell line). The augmentation of enzyme activity increased the amount of formazan dye, which was quantified using a microplate reader (Bio-Rad, Tokyo, Japan) by measuring absorbance at 450 nm. This procedure was repeated at least three times.

### Tissue immunohistochemistry and immunofluorescence

Formalin-fixed paraffin-embedded (FFPE) tissue specimens were sliced at 4 µm thickness, deparaffinized in xylene, and rehydrated using graded ethanol solutions. After activation in citric acid buffer at 98 °C for 40 min, the slides were stained with anti-NNMT (Santa Cruz, Tokyo, Japan G-4; 1:100) antibodies and processed using the EnVision FLEX Visualization System (Agilent Technologies). Samples were counterstained with Mayer’s hematoxylin (131–09665, Fujifilm, Tokyo, Japan).

### Tissue microarray analysis

We analyzed NNMT expression using a tissue microarray (TMA) for 39 patients with ovarian clear cell carcinoma who underwent surgery at Teikyo University Hospital between January 2003 and December 2012. Detailed information about the clinical characteristics was obtained after a retrospective review of the medical records. NNMT immunohistochemical reactivity was scored without knowledge of the clinical outcomes by two observers (R.K. and Y.S.). Each sample was scored based on the percentage of positive cells in each compartment (0, no staining; 1, < 30%; 2, 30%–50%; 3, ≥ 50%). The staining intensity was similar across all samples. Expression was considered ‘low’ if the cytoplasmic staining intensity was 0 or 1 and ‘high’ if scores of 2 or 3 were obtained. The analysis was limited to patients with ovarian clear cell carcinoma (*N* = 39).

### Fluorescence-labeled two-dimensional differential gel electrophoresis (2D-DIGE)

Proteins were labeled using the CyDye DIGE Fluor minimal dye (GE Healthcare), as per the manufacturer’s instructions.

Briefly, 50 mg of a sample mixture extracted from the ovarian clear cell cancer cell lines (OVTOKO, OVMANA, ES-2, and KK) was adjusted to pH 8.5 using 50 mM NaOH, and samples were labeled with 400 pmol Cy5 as a control or with 400 pmol Cy3. Fluorescence labeling was performed on ice in the dark for 30 min, and the reaction was subsequently quenched with 1 mL of 10 mM lysine (Sigma-Aldrich, St. Louis, USA) for 10 min. Each preparation was treated with two sample buffers containing 7 M urea, 2 M thiourea, 4% CHAPS, 1% immobilized pH gradient (IPG)-buffer with a pH range of 4–7, and 2% dithiothreitol (DTT) according to the manufacture’s recommendations. The final volume was adjusted to 260 mL using rehydration buffer (7 M urea, 2 M thiourea, 4% CHAPS, 0.5% IPG buffer with a pH range of 4–7, and 0.2% DTT). The mixture of proteins labeled with Cy3 and Cy5 was applied to Immobiline DryStrips (pH 4–7, 13 cm) (Cytiva, Tokyo, Japan) and focused on a PROTEAN i12 IEF system (Bio-Rad, Tokyo, Japan). Focused IPG strips were equilibrated and loaded onto 12.5% SDS–polyacrylamide gels (30% acrylamide, 1.5 M Tris–HCl pH 8.8, 10% SDS, 10% ammonium persulfate, and 10% TetraMethylEthyleneDiamine (TEMED)) using low-fluorescence glass plates on an SE 600 Ruby system (GE Healthcare). All electrophoresis procedures were performed in the dark. After SDS-PAGE, gels were scanned using a Pharos FX System (Bio-Rad) with appropriate excitation/emission wavelengths specific for Cy3 (532/605 nm) and Cy5 (635/695 nm). Scanned images were analyzed using the PDQuest Advanced Version 8.0 software (Bio-Rad).

PDQuest was used to identify spots with higher or lower protein expression in ES-2 and KK, and OVMANA and OVTOKO, respectively. Five spots were identified. Mass spectrometric analysis showed that NNMT was present in one of the spots. The spot containing NNMT had higher protein levels in OVMANA and OVTOKO than in KK and ES-2. For further confirmation, ES-2 and OVMANA, ES-2 and OVTOKO, KK and OVMANA, KK, and OVTOKO were labeled with Cy3, Cy5, Cy5, and Cy3, respectively. The protein expression level of the spot containing NNMT was higher in OVMANA than ES-2, OVTOKO than ES-2, OVMANA than KK, and OVTOKO than KK.

### Peptide mass fingerprinting (PMF)

All chemicals used in this study were of analytical grade. The compounds 4-Sulfophenyl isothiocyanate, a-cyano-4-hydroxycinnamic acid (CHCA), sodium bicarbonate, and ammonium bicarbonate were purchased from Sigma (St. Louis, MO, USA).

For protein identification by peptide mass fingerprinting, protein spots were excised, digested with trypsin (Promega, Madison, WI), mixed with α-cyano-4-hydroxycinnamic acid in 50% acetonitrile /0.1% TFA, and subjected to matrix-assisted laser desorption/ionization-time of flight (MALDI-TOF) analysis (Microflex LRF 20, Bruker Daltonics, MA, USA), as described by Fernandez et al. (Electrophoresis 19:1036-1045). Spectra were collected from 300 shots per spectrum over an *m/z* range of 600–3000 and calibrated by two-point internal calibration using trypsin auto-digestion peaks (*m/z* 842.5099 and 2211.1046). The peak list was generated using Flex Analysis 3.0. The threshold used for peak-picking was as follows: 500 for the minimum resolution of monoisotopic mass and 5 for S/N. The search program MASCOT, developed by Matrixscience (http://www.matrixscience.com/), was used for protein identification using peptide mass fingerprinting. The following parameters were used for the database search: trypsin as the cleaving enzyme, maximum of one missed cleavage, iodoacetamide (Cys) as a complete modification, oxidation (Met) as a partial modification, monoisotopic masses, and a mass tolerance of ± 0.1 Da. The PMF acceptance criterion included the probability scores.

### Western blot analysis

Equal amounts of protein were fractionated by sodium dodecyl sulfate–polyacrylamide gel electrophoresis and transferred onto a polyvinylidene difluoride membrane (Millipore, Bedford, MA, USA). The membranes were blocked, the primary antibodies were added, and the membranes were incubated with secondary antibodies. Signals were detected using an Image Quant LAS 4000 Mini instrument (GE Healthcare, Wauwatosa, WI, USA).

### siRNA

We transfected NNMT siRNA (sc-61213; Santa Cruz Biotechnology) into the ovarian clear cell carcinoma cell lines (OVTOKO, RMG1, and ES-2) cultured in 6-well plates using Lipofectamine RNAi MAX Reagent (13,778; Invitrogen, Tokyo, Japan) and Opti-MEM Reduced Serum Medium (31,985,070; Thermo Fisher Scientific, Tokyo, Japan). Control siRNA-A (sc-37007; Santa Cruz Biotechnology, Tokyo, Japan) was used as a negative control. The knockdown effect was tested by western blotting using an anti-NNMT antibody.

### Expression plasmid transfection

We transfected the NNMT Expression Plasmid DNA (RC200641; OriGene Technologies, Inc., MD, USA) into the ES-2 cell line that was cultured in 6-well plates using Lipofectamine 2000 Transfection Reagent (11,668,019; Thermo Fisher Scientific) and Opti-MEM Reduced Serum Medium (31,985,070; Thermo Fisher Scientific Tokyo, Japan). The pCMV vector was used as a negative control for all experiments. The cells were incubated for 2 weeks in a medium containing G418 (1000 μg/mL; Thermo Fisher Scientific, Tokyo, Japan). We also established stable control clones expressing NNMT in these cell lines. Drug-resistant clones were further incubated in a medium with puromycin (58-58-2; Sigma-Aldrich, Tokyo, Japan) and tested for the knockdown effect by western blotting using the NNMT antibody.

### Statistical analysis

The Student *t*-test was used to determine the statistical significance of the differences between the comparison groups in vitro. Error bars represent the mean ± standard error of the mean. The relationship between NNMT expression and clinicopathological characteristics was analyzed using Pearson’s *χ2* test. Data are equally distributed, and survival rates were calculated using the Kaplan–Meier method and log-rank test.

## Results

### Proliferation of OCCC cell lines was differentially inhibited by paclitaxel

**Fig. 1:**
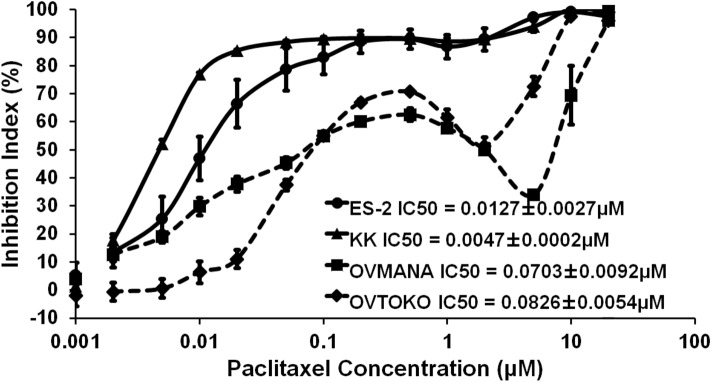
WST-8 assay of 4 ovarian clear cell cancer cell lines. Inhibition rate of the number of viable cells in the ovarian clear cell carcinoma (OCCC) cell lines, OVTOKO, OVMANA, ES-2, and KK assessed at indicated paclitaxel concentrations via the WST-8 assay. IC50 for OVTOKO and OVMANA was higher than IC50 for KK and ES-2. The number of independent experiments is 3

Using the WST-8 assay, we showed that paclitaxel inhibited the proliferation of OVTOKO, OVMANA, ES-2, and KK cells in a dose-dependent manner. Paclitaxel inhibited the proliferation of OVTOKO and OVMANA cells more than that of ES-2 and KK (IC50 = 0.0826 ± 0.0054, 0.0703 ± 0.0092, 0.0127 ± 0.0027, 0.0047 ± 0.0002, respectively) (Fig. 1).

### 2D-DIGE analysis of OCCA cell lines and mass spectrometric analysis

The mixture of OVTOKO, OVMANA, KK, and ES-2 cellular proteins was labeled with Cy5 and used as an internal control. The OVTOKO, OVMANA, KK, and ES-2 proteins from individual cell lines were labeled with Cy3. The mixture of the proteins labeled with Cy3 and Cy5 was analyzed using four 2D-DIGE gels. A total of 130 protein spots were detected from the mixture of proteins from OVTOKO, OVMANA, KK, and ES-2 cells on each gel using PDQuest Advanced software, and all spots on each gel were matched. A total of 128 protein spots were detected, and the spots for OVTOKO, OVMANA, KK, and ES-2 cells, and all spots on each gel were matched using the PDQuest Advanced software.

**Fig. 2:**
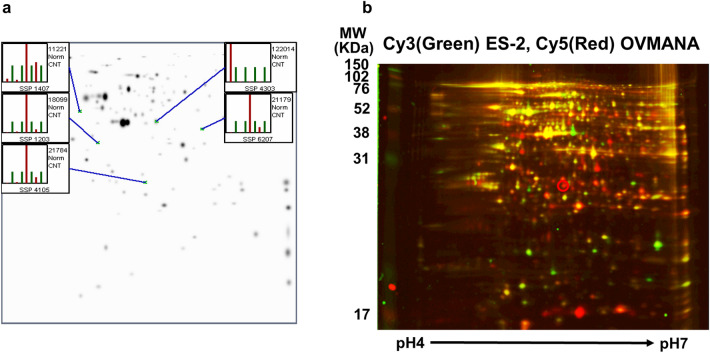
The result of fluorescence-labeled two-dimensional differential gel electrophoresis (2D-DIGE). **a** Protein spots and quantity graph. Proteins extracted from OVTOKO, OVMANA, ES-2, and KK cells were separated using 2D-DIGE and the protein expression changes were analyzed using the PDQuest software. Image analysis using PDQuest software identified 5 protein spots with significantly higher or lower expression in OVTOKO and OVMANA than that in ES-2 and KK cells. **b** A representative gel image of 2D-DIGE is shown. The protein from ES-2 and OVMANA cells were labeled with Cy3 (green) and Cy5 (red), respectively. The red protein spot contained NNMT and its expression was higher in OVMANA than in ES-2 cells. The number of independent experiments is 3

**Fig. 3:**
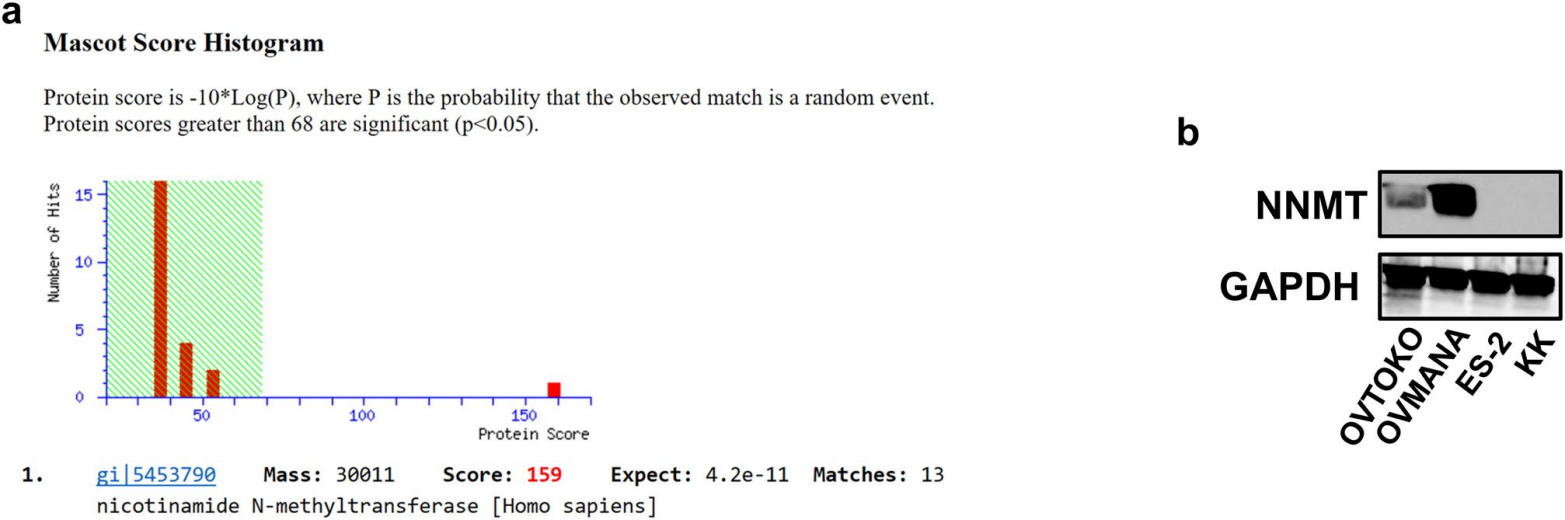
Mascot search result and western blot analysis. **a** The proteins identified using the MASCOT peptide mass fingerprinting server in one of the 5 protein spots. MASCOT analysis showed a top score of 159 for nicotinamide N-methyltransferase (NNMT). The number of independent experiments is 1. **b** Western blot analysis showed that NNMT was expressed in OVTOKO and OVMANA, but not in ES-2 and KK cells. The number of independent experiments is 3

**Fig. 4:**
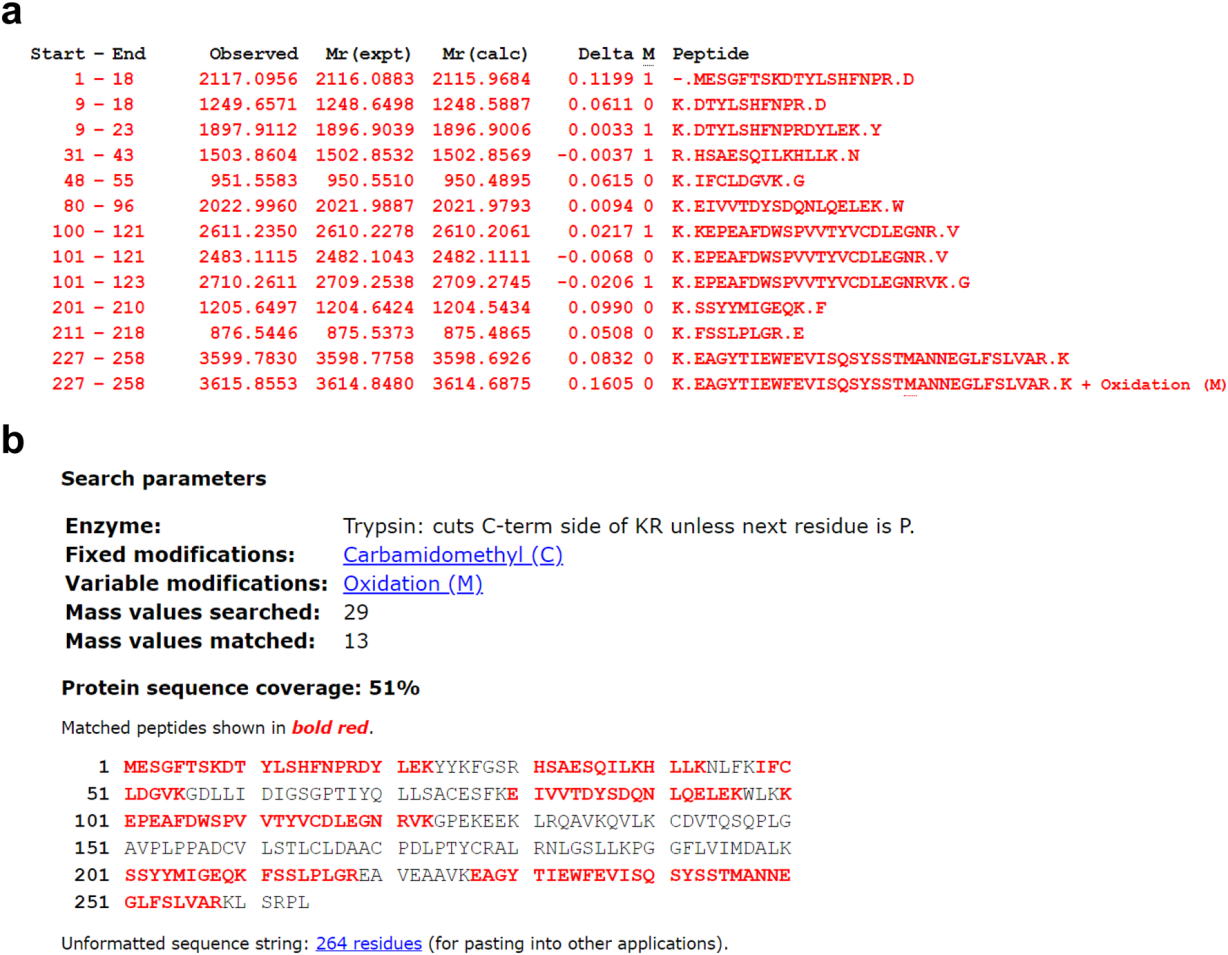
A list of the digested peptides and NNMT protein sequence. **a** A list of the digested peptides that covered the NNMT protein sequence. The number of independent experiments is 1. **b** The digested peptides covered 51% of the NNMT protein sequence. The number of independent experiments is 1

**Fig. 5:**
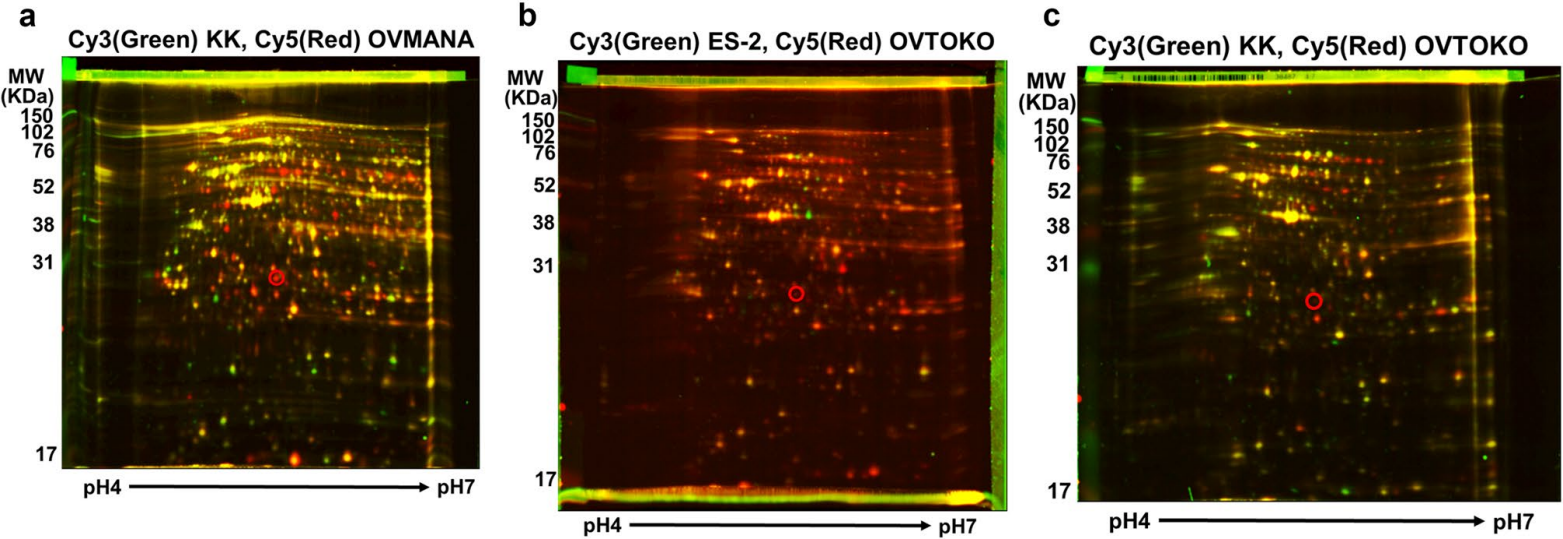
The result of fluorescence-labeled two-dimensional differential gel electrophoresis (2D-DIGE). A representative gel image of 2D-DIGE is shown. **a** The protein from KK and OVMANA cells were labeled with Cy3 (green) and Cy5 (red), respectively. The red protein spot contained NNMT and its expression was higher in OVMANA than in KK cells. The number of independent experiments is 2. **b** The protein from ES-2 and OVTOKO cells were labeled with Cy3 (green) and Cy5 (red), respectively. The red protein spot contained NNMT and its expression was higher in OVTOKO than in ES-2 cells. The number of independent experiments is 2. **c** The protein from KK and OVTOKO cells were labeled with Cy3 (green) and Cy5 (red), respectively. The red protein spot contained NNMT and its expression was higher in OVTOKO than in KK cells. The number of independent experiments is 2

We detected four spots that showed greater intensity in OVTOKO and OVMANA than in KK and ES-2 cells, and one spot that showed greater intensity in KK and ES-2 than in OVTOKO and OVMANA cells by creating analysis sets using PDQuest Advanced software. The spots and quantitative graphs for each spot are shown in Fig. 2a. The red bars in the quantitative graph show the number of spots from ES-2, KK, OVMANA, and OVTOKO cells (from left to right), and the green bars show the number of spots from the mixture of ES-2, KK, OVMANA, and OVTOKO cells on each gel (Fig. 2a). The three spots were detected via Coomassie Brilliant Blue staining. The samples were next subjected to mass spectrometric analysis. In one of the three spots, an unnamed protein with a molecular weight of ~ 36 KDa and a pI of 5.88 was detected in a MASCOT search with the top score of 161 (data not shown). In another one of the 3 spots with a molecular weight of ~ 33 KDa and pI 4.99, we detected Mixture 1 (glyceraldehyde-3-phosphate dehydrogenase, chain A, annexin A2, and chain A, crystal structure of human Mu_crystallin at 2.6 Å) at the top score 350 (data not shown). In another one of the 3 spots with a molecular weight of ~ 30 KDa and pI 5.56, we detected NNMT via a Mascot search with a top score of 159 (Fig. 3a). Figure 4a shows the list of digested peptides derived from the spot, which was identified as NNMT in a MASCOT search. Figure 4b shows the NNMT protein sequence coverage of the digested peptides. We confirmed that the spot containing NNMT had higher protein levels in OVMANA than in ES-2 (Fig. 2b), in OVMANA than in KK, in OVTOKO than in ES-2, and in OVTOKO than in KK cells (Fig. 5a, b, c).

### *Validation of the 2D-DIGE results *via* western blot analysis*

We detected NNMT in OVTOKO and OVMMANA cells, but not in ES-2 and KK cells, using western blot analysis. Thus, the 2D-DIGE results were validated using western blot analysis (Fig. 3b).

### Association between the expression level of NNMT and clinicopathologic characteristics in cases with primary tumors

**Fig. 6:**
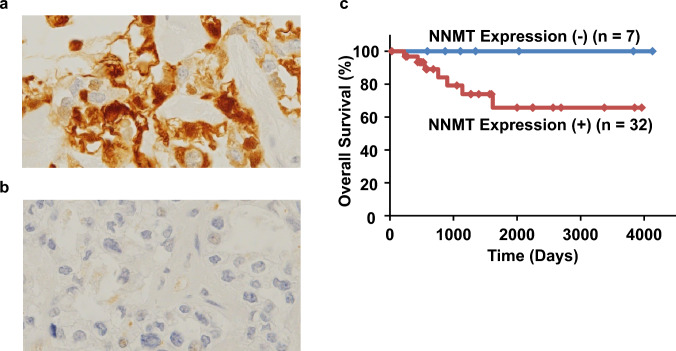
Immunohistochemical staining of NNMT (cytoplasm) and survival analysis of NNMT. Immunohistochemical analysis of NNMT expression in primary OCCC tumors. **a** and **b** Representative NNMT immunohistochemical staining of primary ovarian clear cell carcinoma cells. High (a) or almost no (b) expression of NNMT was observed in the cytoplasm of primary OCCC cells. Magnification, × 400. **c** Kaplan–Meier curve showing the overall survival rates of patients with OCCC. There were no deaths in patients with OCCC, who showed lower NNMT levels in the cytoplasm. The number of samples in the low NNMT expression group was 7, and the number of samples in the high group was 32

To further clarify the clinical significance of NNMT levels in OCCC, the expression level of the protein in primary OCCC tissues was evaluated by immunohistochemistry using an NNMT-specific antibody. Although a few OCCC specimens frequently showed high levels of NNMT in the cytoplasm (Fig. 6a), no or very weak immunoreactivity for NNMT in the cytoplasm was observed in other OCCC specimens (Fig. 6b). The association of cytoplasmic NNMT expression with clinicopathological characteristics of the 39 patients with OCCC is shown in Table 1. Based on the correlation with the clinicopathological characteristics, NNMT expression showed no significant correlation with age, FIGO stage, primary tumor, lymph node metastasis, distant metastasis (UICC classification), and optimal surgery. No deaths occurred in patients with lower levels of NNMT expression in the cytoplasm of tumor cells during the study period, whereas 18% (7 of 39 cases) of patients with higher levels of NNMT expression in the cytoplasm of tumor cells died. The log-rank test showed no significant difference between the two groups (*p* = 0.155), but the hazard ratio (Mantel–Haenszel method) was 3.6, indicating a 3.6-fold higher mortality in the group with NNMT expression (Fig. 6c). A Chi-square test of likelihood ratios for the presence or absence of NNMT expression and whether the patient died or survived showed a trend toward more deaths in the high NNMT expression group than in the low NNMT expression group (*p* = 0.0789). Patients with high NNMT immunoreactivity in the tumor stroma had significantly shorter progression-free survival than those with low NNMT immunoreactivity (*p* = 0.0115, log-rank test) (data not shown).

**Table 1:** Association of NNMT expression with clinicopathological characteristics of 39 ovarian clear cell carcinomas

Patient characteristics	*n*	NNMTh (%)	Pearson’s χ^2^	*P*
Total	39	32 (82.1)		
Age (years)	< 60 (25)	22 (88.0)	1.673	0.196
	≥ 60 (14)	10 (71.4)		
Stage				
Ⅰ	27	22 (81.5)	3.188	0.364
Ⅱ	5	5 (100)		
Ⅲ	5	3 (60.0)		
Ⅳ	2	2 (100)		
Primary tumor				
T1	28	22 (78.6)	1.331	0.514
T2	6	5 (83.3)		
T3	5	5 (100)		
Lymph node metastasis				
N0	35	30 (85.7)	3.109	0.078
N1	4	2 (50)		
Distant metastasis				
M0	37	30 (81.1)	0.461	0.497
M1	2	2 (100)		
Optimal surgery				
Complete surgery	29	23 (79.3)	0.577	0.448
Incomplete surgery	10	9 (90)		

NNMTh high NNMT expression

### Knockdown of NNMT expression in OVMANA and RMG1 cells with NNMT siRNA reduced paclitaxel efficacy

**Fig. 7:**
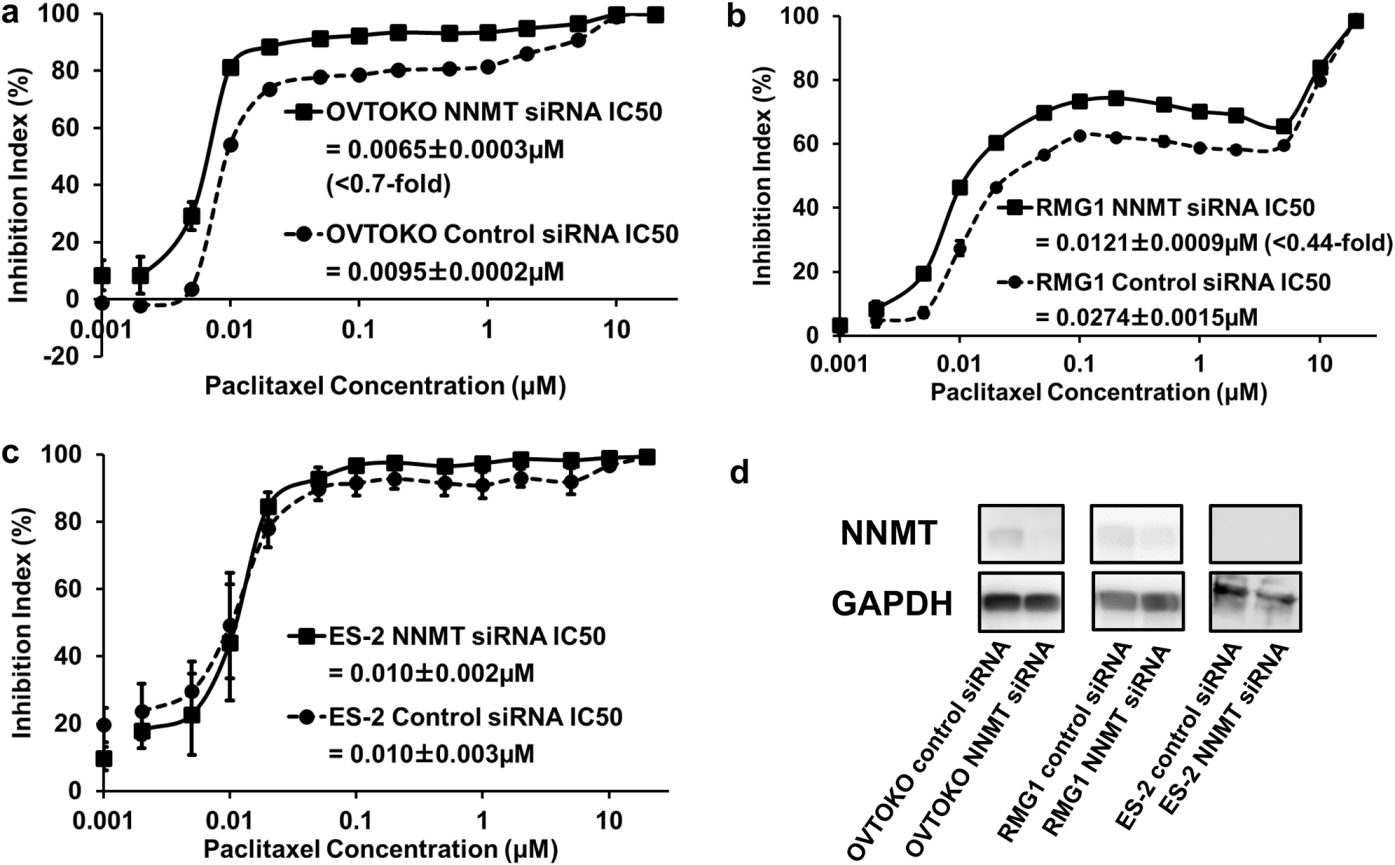
The result of NNMT siRNA and western blot analysis. We investigated changes in paclitaxel sensitivity after NNMT siRNA-dependent knockdown of OVTOKO and RMG1 using the WST-8 assay. The graphs were created using the mean of the three experiments and SEM is shown as error bars. **a** Paclitaxel IC50 in the NNMT siRNA-transfected OVTOKO cells is significantly lower than that of the control siRNA-transfected OVTOKO cells (< 0.7-fold, *p* = 0.00275, student t-test). The number of independent experiments is 3. **b** Paclitaxel IC50 in the NNMT siRNA-transfected RMG1 cells is significantly lower than that of the control siRNA-transfected RMG1 cells (< 0.44-fold, *p* = 0.00190, student t-test). The number of independent experiments is 3. **c** Paclitaxel IC50 in the NNMT siRNA-transfected ES-2 cells is not significantly lower than that of the control siRNA-transfected ES-2 cells (1.01-fold, *p* = 0.984, student t-test). The number of independent experiments is 3. **d** Western blot analysis showed reduced expression of NNMT protein in OVTOKO and RMG1 cells in the NNMT siRNA-treated group compared to the control siRNA-treated group. No difference was observed between the two groups in ES-2 cells because of the absence of NNMT expression. The number of independent experiments is 3

NNMT was knocked down using siRNA, and the decrease in NNMT protein expression was confirmed by western blotting (Fig. 7d). Cell proliferation was examined using a WST-8 assay. The IC50 of OVTOKO cells with siRNA knockdown of NNMT was lower than the IC50 of cells treated with control siRNA (< 0.7-fold, *p* = 0.00275, student *t*-test) (Fig. 7a). The doubling times of OVTOKO cells knocked down with NNMT siRNA and OVTOKO cells treated with control siRNA were 25.1 h and 27.2 h, respectively, with no significant difference in doubling time (*p* = 0.295, paired *t*-test). In addition, the IC50 of RMG1 cells with siRNA knockdown of NNMT was lower than the IC50 of cells treated with control siRNA (< 0.44-fold, *p* = 0.00190, student *t*-test) (Fig. 7b). The doubling times of RMG1 cells knocked down with NNMT siRNA and RMG1 cells treated with control siRNA were 22.0 h and 22.6 h, respectively, with no significant difference in doubling time (*p* = 0.906, paired *t*-test). Using ES-2 cells without NNMT expression, we examined whether cell proliferation differed between the NNMT and the control siRNA-treated groups. Cell proliferation was similar in both groups (*p* = 0.984, student *t*-test) (Fig. 7c). The doubling times of ES-2 cells knocked down with NNMT siRNA and ES-2 cells treated with control siRNA were 97.7 h and 66.0 h, respectively, with no significant difference in doubling time (*p* = 0.0872, paired *t*-test).

### Overexpression of NNMT in ES-2 cells did not alter their sensitivity to paclitaxel

**Fig. 8:**
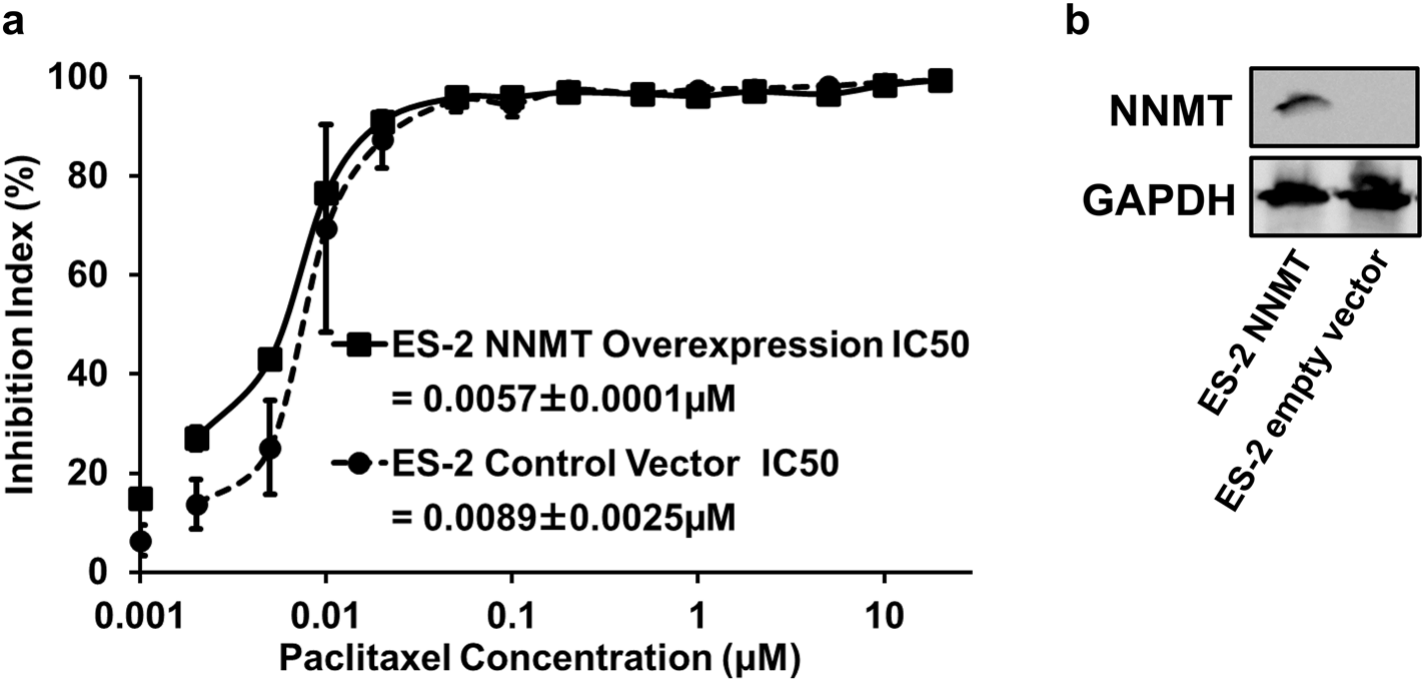
The result of NNMT overexpression vector and western blot analysis. We investigated changes in paclitaxel sensitivity after NNMT overexpression in ES-2 cells using the WST-8 assay. **a** Paclitaxel IC50 in the NNMT-overexpressing viable ES-2 cells was not significantly higher than that of ES-2 transfected with empty vector (pCMV vector) (*p* = 0.360, student t-test). The number of independent experiments is 3. **b** Western blot analysis showed the increased expression of NNMT protein in ES-2 cells in the NNMT overexpression group compared to the empty vector-transfected group. The number of independent experiments is 3

ES-2 cells were used to create a stable cell line overexpressing NNMT, and the increase in NNMT protein was confirmed by western blotting (Fig. 8b). The NNMT-overexpressing line of ES-2 cells showed no change in proliferative ability after paclitaxel treatment compared to the control vector (pCMV vector)-transfected line (*p* = 0.360, student *t*-test) (Fig. 8a). The doubling times of ES-2 cells overexpressing NNMT and cells transfected with the control vector (pCMV vector) were 43.0 h and 41.8 h, respectively, with no significant difference (*p* = 0.894, paired *t*-test).

## Discussion

Ovarian cancer is the fifth most common cancer-related death in the USA, with 12,810 women dying from it annually [1]. OCCC is common in Asia; however, patients with advanced or recurrent disease have poor survival outcomes. Owing to intrinsic chemotherapy resistance, OCCC shows reduced sensitivity to chemotherapy [2]. Treatment failure due to chemotherapy resistance in advanced or recurrent cancers is a significant dilemma in the treatment of OCCC. Therefore, the search for indicators that can predict the efficacy of chemotherapy may improve the prognosis of patients with OCCC. Several biomarkers in tumor tissues have been used to predict the efficacy of chemotherapeutic drugs in ovarian cancer research and clinical settings. Demethylation of HIN-1 reverses paclitaxel resistance in OCCC through the AKT-mTOR signaling pathway [7]. Everolimus, administered after 5-aza-2-deoxycytidine treatment, is a promising therapy for paclitaxel-resistant OCCC and targets AKT/mTOR pathway and EZH2 factor [14]. A global proteomic study has been performed in 192 cases of OCCC, and the analysis has allowed us to separate the lipid metabolic activity cluster and the coagulation cluster. Accumulation of fibrinogen subunits in tumors within coagulation clusters may reflect the risk of developing deep vein thrombosis. Of particular interest is the increased lipid metabolic processes characteristic of OCCC, where the pale cytoplasm contains fat droplets in addition to glycogen, which may act as an alternative fuel for tumor growth, and findings suggesting an aggressive response to oxidative damage have also been observed [15].

However, more reliable biomarkers are required to predict the efficacy of chemotherapeutic agents against all OCCC molecular subtypes during treatment. In this study, we identified higher NNMT expression in the OVTOKO and OVMANA cell lines that showed reduced paclitaxel sensitivity than ES-2 and KK cells. NNMT is predominantly expressed in the liver and catalyzes the N-methylation of nicotinamide, pyridines, and other structural analogs involved in the biotransformation and detoxification of several drugs and xenobiotic compounds. To the best of our knowledge, there have been no direct studies on NNMT expression in OCCC. However, NNMT protein is overexpressed in renal clear cell carcinoma (ccRCC) compared to non-tumor tissue, and high NNMT expression in primary ccRCC has been associated with poor prognosis in an independent cohort. [16] In addition, NNMT inhibitors (NNMTi) alone or in combination with 2-deoxy-D-glucose and BPTES have been reported to inhibit cell survival in ccRCC cell lines and primary tumor and metastatic models, suggesting that NNMT inhibitors may provide a new therapeutic strategy for ccRCC and function as a sensitizer for combination therapy [16]

OCCC and ccRCC are characterized by the accumulation of glycogen in the cytoplasm, resulting in a distinct cytoplasmic appearance. Genomic studies have shown multiple similarities in mutations between these two diseases, including frequent similarities in mutations in the lipid-3 kinase-mammalian target of rapamycin (mTOR) pathway involved in cell proliferation [17]. Pharmacological activation of AMP-activated protein kinase (AMPK), an intracellular energy sensor, and inhibition of the mTOR pathway have been reported to increase NNMT levels [18]. Growing evidence has shown that NNMT is aberrantly expressed in several cancers and is a promising prognostic predictor in cancers such as gastric carcinoma, oral squamous cell carcinoma, ovarian cancer, hepatocellular carcinoma, and nasopharyngeal carcinoma [19–23]. NNMT expression reduces sensitivity to anticancer drugs in several cancer cell types. For example, NNMT expression reduced apoptosis induced by adriamycin or paclitaxel in breast cancer cells [24]. Further, NNMT reduces sensitivity to 5-fluorouracil in colorectal cancer cells [25]. Thus, the role of NNMT in various carcinomas has evolved from a mere metabolic function to being a driving force in diseases, including various cancers. However, despite growing evidence that NNMT is an effective therapeutic target, no cell-active inhibitors of this enzyme have been developed. Expression in cancer-associated fibroblasts (CAFs) in highly atypical serous carcinomas in ovarian cancer, NNMT expression in CAFs, has resulted in depletion of S-adenosylmethionine and decreased histone methylation associated with extensive gene expression changes in the tumor stroma [26].

Here, we investigated the effects of NNMT on paclitaxel resistance in OCCC cells. The OVTOKO and RMG1 cell lines, which show high NNMT expression, and the ES-2 cell line, which shows no NNMT expression, were selected for this study. NNMT knockdown increased paclitaxel sensitivity in OVTOKO and RMG1 cells, but not in ES-2 cells. Conversely, NNMT overexpression in ES-2 cells did not alter paclitaxel sensitivity. These results indicate that altering endogenous NNMT levels may affect paclitaxel sensitivity. NNMT inhibitors [27] may be used in the future to increase drug sensitivity in paclitaxel-resistant cancers. Cytoplasmic expression of NNMT may worsen the prognosis of OCCC. In high-grade serous ovarian carcinoma, NNMT expression in the tumor stroma is involved in the differentiation of cancer-associated fibroblasts, suggesting a potential role in cancer progression in the stroma [21, 25]. However, NNMT expression in OCCC tumor cells may be involved in the association between chemotherapy sensitivity and prognosis. NNMT expression in ovarian cancer cells is associated with poor prognosis [21]. Although there are no reports in ovarian cancer, one study showed that NNMT expression in breast cancer tumor cells enhances chemotherapy resistance [24]. In our study, NNMT showed potential to serve as a biomarker for the prediction of diagnostic and chemotherapeutic efficacy in OCCC. In OCCC with NNMT expression, suppression of intrinsic NNMT expression may increase paclitaxel sensitivity. These results indicated that NNMT may be a potential therapeutic target for OCCC. However, the exact mechanism by which NNMT regulates paclitaxel sensitivity requires further investigation.

## Conclusions

Our study demonstrated for the first time that NNMT is overexpressed in OCCC and high NNMT expression levels may correlate with poor survival outcomes and an unfavorable therapeutic response in patients who received chemotherapy. Further, reduced NNMT expression enhanced the sensitivity of OCCC cells to paclitaxel-induced cell death. Taken together, these results suggest that NNMT is a promising new therapeutic target for OCCC.

## Data Availability

The data that support the findings of this study are available from the corresponding author, RK, upon reasonable request.
